# Rapid Quantitative Assessment of Muscle Sodium Dynamics After Exercise Using ^23^Na‐MRI in Dysferlinopathy and Healthy Controls

**DOI:** 10.1002/jcsm.13709

**Published:** 2025-02-03

**Authors:** Mary A. Neal, Carla F. Bolano‐Diaz, Mark Richardson, Jassi Michell‐Sodhi, Robert Muni‐Lofra, Meredith K. James, Kieren G. Hollingsworth, Heather Hilsden, Ian Wilson, Andrew M. Blamire, Volker Straub, Peter E. Thelwall, Jordi Diaz‐Manera

**Affiliations:** ^1^ Translational and Clinical Research Institute Newcastle University Newcastle upon Tyne UK; ^2^ Newcastle Magnetic Resonance Centre, Health Innovation Neighbourhood Newcastle University Newcastle upon Tyne UK; ^3^ The John Walton Muscular Dystrophy Research Centre, Translational and Clinical Research Institute Newcastle University and Newcastle Hospitals NHS Foundation Trust Newcastle upon Tyne UK; ^4^ Newcastle Hospitals NHS Foundation Trust Newcastle upon Tyne UK; ^5^ Laboratori de Malalties Neuromusculars Insitut de Recerca de l'Hospital de la Santa Creu i Sant Pau Barcelona Spain; ^6^ Centro de Investigación en Red en Enfermedades Raras (CIBERER) Instituto de Salud Carlos III Barcelona Spain

**Keywords:** biomarkers, dysferlinopathy, imaging, muscle MRI, muscular dystrophy, sodium MRI

## Abstract

**Background:**

Dysferlin plays a key role in cell membrane repair; its absence or malfunction in patients with dysferlin‐deficient limb girdle muscular dystrophy leads to muscle fibre death. Muscle magnetic resonance (MR) imaging allows non‐invasive and repeatable measurements that can report on pathological changes observed in dysferlinopathy patients (DP). We aimed to demonstrate the feasibility of utilising volume‐localised ^23^Na spectroscopy as a novel approach to characterise muscle Na^+^ content and biexponential T_2_* at rest, and dynamically post‐exercise, in patients with dysferlinopathy and in matched healthy controls.

**Methods:**

Adult DP and age and sex matched healthy volunteers (HV) were recruited and scanned on a 3 T clinical MR scanner. Following baseline scans, participants performed physiotherapist‐guided isometric dorsiflexion contractions until *tibialis anterior* (TA) muscle exhaustion. Dynamic volume‐localised sodium‐23 (^23^Na)‐ and proton (^1^H)‐MR scans were acquired serially for 35 min post‐exercise. MR data were analysed to determine TA lipid content, change in TA sodium content, biexponential sodium T_2_* properties and TA water ^1^H T_2_.

**Results:**

Ten DP (mean age ± standard deviation [SD]: 38.0 ± 10.8 years; 80% female) and 10 HV (mean age ± SD: 38.9 ± 11.5 years) were scanned. Baseline muscle water ^1^H T_2_ and sodium concentration were significantly higher in DP compared to matched controls (^1^H T_2 DP_ [SD] = 33.8 [2.7] ms, ^1^H T_2 HV_ = 29.3 [1.1] ms, *p* < 0.001; [^23^Na]_DP_ = 36.2 [11.4] mM, [^23^Na]_HV_ = 19.6 [3.1] mM, *p* < 0.001). ^1^H T_2_ and sodium content in healthy controls showed significant post‐exercise elevation with a slower time‐to‐peak for sodium content compared to ^1^H T_2_. ^1^H T_2_ and sodium content change post‐exercise was highly variable in the DP group. Notably, ^23^Na dynamics in one DP with normal muscle fat fraction were similar to HV. Biexponential ^23^Na T_2_* was measured at baseline in HV (T_2_*_slow_ = 13.4 [2.3] ms, T_2_*_fast_ = 2.2 [1.3] ms), and DP (T_2_*_slow_ = 14.0 [1.5] ms and T_2_*_fast_ = 1.0 [0.5] m). Equivalent measurements post‐exercise revealed an increase in the fraction of the slow‐relaxing component in HV (*p* < 0.05), consistent with oedematous changes.

**Conclusions:**

Assessment of TA muscle fat fraction, ^1^H T_2_, sodium content and sodium T_2_* relaxation properties revealed differences at baseline and in post‐exercise dynamics between patients with dysferlinopathy and matched controls. Post‐exercise ^23^Na recovery dynamics followed a well‐defined time course in HV. Heterogeneous alterations in sodium content and MR relaxation properties in DP may reflect altered ion homeostasis associated with chronic muscle damage.

## 1 Introduction

Dysferlin is a 230‐KDa protein involved in membrane repair of skeletal muscle fibres and the function of the T‐tubule system encoded by the *DYSF* gene. Patients with dysferlin‐deficient limb girdle muscular dystrophy develop progressive muscle weakness with variable presentations [1]. Muscle weakness typically becomes more generalised as the disease progresses, leading to severe and irreversible disability [2, 3].

Muscle degeneration in muscular dystrophies progresses in a stepwise process. In a first stage, the absence or malfunction of a muscle protein (such as dysferlin) induces intracellular changes that result in muscle fibre damage. This leads to the second stage of muscle inflammation and fibre necrosis. In the third stage, these damaged muscle fibres are substituted by fat and fibrotic tissue, which are unable to contract, resulting in muscle weakness and disability.

Magnetic resonance imaging (MRI) provides non‐invasive methods capable of reporting on these stages of disease progression. Conventional MRI maps the distribution of hydrogen (^1^H) nuclei in tissue water and fat, with contrast in resultant images influenced by the T_1_ and T_2_ magnetic relaxation rates of ^1^H nuclei. T_2_‐weighted images are sensitive to oedema and reduction in cellularity associated with inflammation and necrosis. T_2_ relaxation rates of tissue water are typically prolonged in skeletal muscle of patients with dysferlinopathy, even at early stages of the disease [4] and, as in other muscular dystrophies, increased T_2_ relaxation time is interpreted as a marker of disease activity. In dysferlinopathy patients, the use of T_2_ as a predictor of disease progression has been proposed, albeit with low sensitivity and specificity [5]. Increased muscle fat content can be visualised by a decrease in muscle T_1_, visible as higher signal intensity in T_1_‐weighted images. Accurate quantitation of muscle fat content can be achieved by the Dixon method [6], which is in current use as an outcome measure in several natural history studies and clinical trials [7, 8]. Muscle fat content correlates with muscle function and forms a good biomarker of disease progression, though as it reports on loss of functional muscle fibres its ability to *predict* changes over time is limited [9].

Other biologically relevant nuclei can be detected with suitably equipped clinical MRI scanners, including phosphorus (^31^P), carbon (^13^C) and sodium (^23^Na). Multinuclear MR enables measurement of metabolite and ion dynamics in body tissues. ^31^P MR spectroscopy has been applied to dysferlinopathy, showing increased turnover of muscle membrane components and a decreased energy production in skeletal muscles of patients [4]. Such measurements have potential sensitivity to earlier stages of dysferlinopathy progression.

Similarly, ^23^Na‐MR can provide metrics of normal and dysfunctional muscle function. Sodium (Na^+^) and potassium (K^+^) ion concentrations are markedly different between the extracellular and intracellular space due to Na^+^/K^+^‐ATPase pumps located in the muscle fibre membrane generating trans‐membrane ion gradients. At rest, the Na^+^ concentration is approximately 10–30 mM in the intracellular and 145 mM in the extracellular compartments, whereas the K^+^ concentration is approximately 140 mM in the intracellular and 4 mM in the extracellular compartments. When muscle contraction takes place, there is an influx of Na^+^ and an efflux of K^+^ ions in the intracellular space [10]. Chronic damage to striated muscle fibres following an acute injury leads to a sustained increase in the resting intracellular levels of Na^+^, though the mechanism behind this has not been completely elucidated (increase in the influx, decrease in the efflux, malfunctioning of the Na^+^/K^+^‐ATPase pumps, membrane integrity and permeability or a combination of factors) [11, 12].

^23^Na‐MRI has been used to study skeletal muscle in both healthy controls and people with muscle disorders, at rest and after exercise [10, 13, 14, 15, 16], using gradient echo and radial/ultrashort echo (UTE) ^23^Na‐MRI scan sequences. ^23^Na nuclei exhibit more complex magnetic relaxation properties than seen with ^1^H nuclei in conventional MRI, with T_2_* relaxation comprising fast‐ and slow‐relaxing components in biological tissues. ^23^Na T_2_* relaxation is entirely slow‐relaxing in dilute aqueous solutions, whereas in homogenous macromolecule‐dense environments its T_2_* relaxation exhibits biexponential relaxation with a 60:40 split between fast and slow relaxation rates [17]. Therefore, we are not only able to measure the intensity of the signal arising from the ^23^Na nuclei but also their probable distribution across cellular environments [18, 19, 20]. This sensitivity of T_2_* relaxation to tissue environment thus introduces complexities into ^23^Na‐MRI quantification (largely mitigated by use of UTE scans) [21]. Dynamic measurements of tissue Na^+^ content and distribution have temporal resolution limited by the duration of ^23^Na‐MRI scans, which take longer to acquire and/or have lower spatial resolution than conventional ^1^H‐MRI. We report in this study ^23^Na‐MR measurements of human post‐exercise Na^+^ dynamics at higher temporal resolution than previously reported by using volume‐localised ^23^Na spectroscopy instead of ^23^Na‐MRI. This approach permitted characterisation of muscle Na^+^ content at rest, and post‐exercise Na^+^ dynamics, in patients with dysferlinopathy and in matched controls. These measurements were coupled with dynamic ^1^H‐MR spectroscopy measurements of tissue water T_2_ and ^1^H‐MRI measures of tissue composition, targeting detection of upstream (Na^+^ homeostasis) and downstream (muscle replacement by fat) processes in dysferlinopathy muscle damage and degeneration. These repeatable and non‐invasive muscle pathology measures hold value as biomarkers in the development of novel therapeutic approaches for dysferlinopathy that aim to restore expression of dysferlin.

## 2 Methods

### 2.1 Study Design and Subjects

This single‐centre study was performed in Newcastle upon Tyne, United Kingdom, as part of the Clinical Outcome Study for Dysferlinopathy (COS‐2), funded by the Jain Foundation. COS‐2 is a natural history study collecting prospective longitudinal clinical and radiological data in patients with a genetic diagnosis of dysferlinopathy. This substudy performed dynamic ^23^Na‐ and ^1^H‐MRI measurements in a subset of COS‐2 participants and in healthy volunteers, under approvals granted by the UK National Health Service Health Research Authority (IRAS project ID 85750) and granted by the Newcastle University Research Ethics Committee (REC reference number 11/NE/0360), in compliance with the ethical principles derived from the Declaration of Helsinki. Healthy volunteers were recruited from the staff and student population within our institution. Written informed consent was obtained from all participants prior to study inclusion. Ten patients with dysferlinopathy and 10 individually age and sex matched healthy volunteers were recruited between September 2022 and May 2023. Patients were aged 18 years or older and were able to achieve at least neutral position against gravity when dorsiflexing the assessed foot on the day of the scan session. Healthy volunteers had no history of neuromuscular diseases or any other disorder that could limit their mobility, with full motion in ankle dorsiflexion.

### 2.2 MRI Acquisition

**FIGURE 1:**
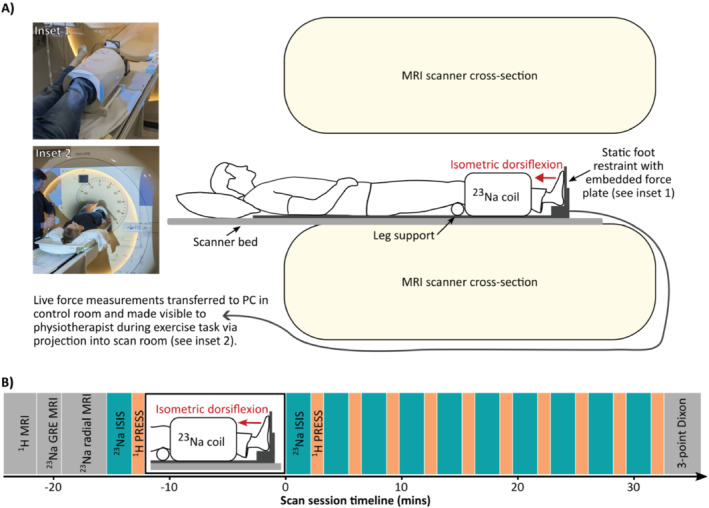
Overview of study protocol. (A) Illustration of participant positioning during exercise and MRI scans. (B) Representative timeline of MRI scan session. Dynamic ^23^Na acquisitions (turquoise) and dynamic ^1^H acquisitions are performed at least once at baseline and alternately at least 10 times post‐exercise. The 3‐point Dixon acquisition was acquired on a separate day in the patient cohort. ^1^H: hydrogen‐1, ^23^Na: sodium‐23, GRE: gradient echo, ISIS: image selected in vivo spectroscopy, mins: minutes, MRI: magnetic resonance imaging, PRESS: point resolved spectroscopy.

The study investigated the *tibialis anterior* (TA) muscle. MR data were acquired using a Philips 3T Achieva scanner (Philips Healthcare, UK) equipped with a 20‐cm diameter ^23^Na quadrature birdcage coil (Rapid Biomedical GmbH, Germany). All participants were positioned feet‐first supine on the scanner bed and the ^23^Na coil positioned with its centre at the widest part of the calf of the stronger leg. The stronger leg was identified by the participant on the day of the study session. For healthy volunteers, the dominant leg was used. Four aqueous Na^+^ concentration standards were constructed, containing 10‐, 20‐, 30‐ and 40‐mM NaCl and 3% agarose (w/v, Sigma‐Aldrich, UK) in 50‐mL cylindrical containers (approx. 50‐mL volume). These standards were positioned within the ^23^Na coil adjacent and immediately anterior to the widest part of the calf. Foam padding was used within the coil volume to immobilise the lower leg and the foot was strapped securely to a rigid foot plate with integral force transducer (Figure 1A).

MRI data were acquired pre‐ and post‐exercise. Table 1 shows scan parameters for the MR sequences employed in this study. High‐resolution multi‐slice axial anatomical ^1^H scans were acquired prior to leg exercise using an out‐of‐phase echo time (TE) to provide clear muscle‐fat delineation. ^23^Na‐MRI scans were spin‐density weighted, acquired with a 2D cartesian gradient echo sequence and with a 3D radial stack of stars sequence, for different T_2_* sensitivity. ^1^H and ^23^Na spectroscopic scans were acquired using PRESS [22] and ISIS [23] volume localisation methods respectively. Spectroscopy scan durations were 45 s (^1^H) and 129 s (^23^Na). Both spectroscopy scans were acquired with the same nominal voxel dimensions (a minimum of 10 × 10 × 50 mm^3^) and location and were positioned entirely within the TA muscle and avoiding major vasculature. The ^23^Na‐ISIS acquisition was repeated three times at baseline in a subset of participants (6 patients and 10 healthy volunteers) to assess inter‐scan repeatability. Participants performed an exercise protocol to induce muscle fatigue whilst positioned in the scanner (described below). A short‐duration (~30s) ^1^H scan was acquired immediately post‐exercise to inform the new voxel position for the subsequent acquisitions in instances where the leg positioning had subtly changed, then ^23^Na‐ISIS and ^1^H‐PRESS acquisitions were acquired in a repeated interleaved fashion for a minimum of 30 min (a minimum of 10 interleaved ^23^Na/^1^H dynamic timepoints) (Figure 1B).

**TABLE 1:** MR sequence acquisition parameters.

	^1^H anatomical	^23^Na GRE	^23^Na radial	^23^Na‐ISIS	^1^H‐PRESS	3‐point Dixon
Summary description of acquisition	Multi‐slice gradient echo with out of phase sequence	2D slice‐selective gradient echo	3D ultrashort echo time radial stack‐of‐stars	Single voxel FID spectroscopy with 3D excitation	Single voxel multi‐echo point‐resolved spectroscopy	Multi‐slice gradient echo, with an ‘out‐in‐out’ phase scheme
RF coil used	Integrated body coil	Rapid quadrature birdcage ^23^Na knee coil	Rapid quadrature birdcage ^23^Na knee coil	Rapid quadrature birdcage ^23^Na knee coil	Integrated body coil	Anterior and posterior 32‐channel phase array coil
FOV (mm^3^)	200 × 200 × 200	192 × 192 × 50	192 × 192 × 225	—	—	410 × 308 × 130
Voxel size (mm^3^)	1 × 1 × 8	6 × 6 × 50	6 × 6 × 25	[10–20] × [10–20] × 50, participant dependent	[10–20] × [10–20] × 50, participant dependent	1 × 1 × 10 (with 20‐mm slice gap)
Signal averages	3	37	400% (radial percentage)	800	1	2
Readout duration (ms)	—	—	—	128	256	—
Samples	—	—	—	1024	1024	—
Slice orientation	Axial	Axial	Axial	Axial	Axial	Axial
TE (ms)	3.5	1.89	0.28	0.16	30, 40, 60, 80, 100, 150	3.45, 4.60, 5.75
TR (ms)	135	100	100	162	6000	100
FA (degrees)	8	90	80	90	90	8
Receiver or spectral bandwidth (Hz/pixel)	434	1773	40.5	8000	4000	433
Acquisition length (min:s)	2:43	1:59	3:51	2:09	0:42	3:05
Acquisition time, inc. preparatory steps (min:s)	—	—	—	2:23	0:54	—

Abbreviations: ^1^H: hydrogen‐1; 2D: two‐dimensional; ^23^Na: sodium‐23; 3D: three‐dimensional; FA: flip angle; FID: free induction decay; FOV: field of view; GRE: gradient echo; Hz: Hertz; inc: including; ISIS: image selected in vivo spectroscopy; min: minutes; mm^3^: cubic millimetres; MR: magnetic resonance; ms: milliseconds; PRESS: point resolved spectroscopy; RF: radiofrequency; s: seconds; TE: echo time; TR: repetition time.

A multi‐slice 3‐point Dixon sequence was performed [6] to assess fat fraction percentage (FF%) within the TA muscle. For the healthy participants, this was acquired as the final scan in the scan session, at a minimum of 30 min after exercise completion, using a 16‐channel torso ^1^H receive array coil (Philips Healthcare, UK). For all patients, this Dixon scan had been acquired as a component of a separate research study visit previously performed at the same centre. Dixon scans were acquired within 2 days of the ^23^Na‐MRI for seven of the 10 patients. Scheduling limitations prevented concurrent ^23^Na‐MRI and Dixon acquisition for the remaining three patients, so data from Dixon scans acquired between 1 and 2 years prior are instead reported.

### 2.3 Exercise Protocol

Leg isometric dorsiflexion exercise was performed after baseline MR acquisitions. Participants were asked to perform a maximum of five sets of maximum‐effort isometric dorsiflexion contractions while lying supine in the scanner. For each exercise set, the participants were requested to contract and hold the contraction of the TA muscle for 30 s 5 times, with a 5‐s rest period between contractions. These sets were repeated with a 30‐s rest between each. Exercise sets continued until participants either completed 5 sets, or the peak force of a set dropped below 50% of that achieved in the initial set, as measured by the force transducer, which provided real‐time feedback to a display visible in the scanning suite. A physiotherapist specialising in neuromuscular disorders was in the scanner room to guide participants through the exercise task, to provide encouragement and to minimise compensatory manoeuvres involving other muscle groups.

### 2.4 Image Analysis

Scan data were anonymised prior to processing and analysis. Muscle MRI data were analysed by a blinded reader who did not have access to participants' clinical information.

The central two slices of the radial ^23^Na‐MRI were summed to produce a slice with 50‐mm thickness with positional equivalence to the cartesian gradient echo ^23^Na scan. Baseline ^23^Na‐MRI signal to noise ratio (SNR) was measured from radial and cartesian ^23^Na‐MRI scan data using a region of interest (ROI) positioned within the TA, and a noise ROI positioned anterior to the leg and phantoms. SNR was calculated using the formula shown in Equation (1).

(1) $$\begin{array}{c} {\mathrm{SNR}}_{\mathrm{TA}}=\frac{\mathrm{mean signal amplitude}-\mathrm{mean noise}}{\mathrm{standard deviation of noise}} \end{array}$$

(2) $$\begin{array}{c} {\left[\mathrm{Na}\right]}^{\mathrm{TA}}={\left[\mathrm{Na}\right]}^{P}\times \frac{{\mathrm{SI}}^{\mathrm{TA}}}{{\mathrm{SI}}^{P}}\times \frac{M_{s}^{P}e^{-\mathrm{TE}/T_{2\;s}^{*P}}+M_{f}^{P}e^{-\mathrm{TE}/T_{2\;f}^{*P}}}{M_{s}^{\mathrm{TA}}e^{-\mathrm{TE}/T_{2\;s}^{*\mathrm{TA}}}+M_{f}^{\mathrm{TA}}e^{-\mathrm{TE}/T_{2\;f}^{*\mathrm{TA}}}} \end{array}$$

The average sodium concentration in the skeletal muscle at baseline was calculated from the radial ^23^Na scan data using the formula shown in Equation (2), where ${\left[\mathrm{Na}\right]}^{P}$ is the concentration in the reference phantom containing 20‐mM NaCl. ${\mathrm{SI}}^{\mathrm{TA}}$ and ${\mathrm{SI}}^{P}$ are the measured ^23^Na‐MRI signal intensities in the TA and the reference phantom respectively. $M_{s}$ and $M_{f}$ are the relative magnitudes of slow‐ and fast‐decaying fractions of ^23^Na signal from the reference phantom or from TA ^23^Na signal, where $M_{s}^{P}+M_{f}^{P}=1$ and $M_{s}^{\mathrm{TA}}+M_{f}^{\mathrm{TA}}=1$. Similarly, $T_{2\;s}^{*}$ and $T_{2\;f}^{*}$ are the slow and fast T_2_* relaxation rates of the signal. $T_{2\;s}^{*P}$ and $T_{2\;f}^{*P}$ were measured from the 20‐mM reference phantom prior to the commencement of this study using an unlocalised ^23^Na free induction decay (acquisition parameters: samples = 1024, spectral bandwidth = 8000 Hz, readout duration = 128 ms, TR = 250 ms, averages = 1000, acquisition length [min:s] = 4:10). $M_{f}^{\mathrm{TA}}$, $M_{s}^{\mathrm{TA}}$, $T_{2\;f}^{*\mathrm{TA}}$, and $T_{2\;s}^{*\mathrm{TA}}$ values were taken from the baseline ^23^Na‐ISIS measurements for each participant, detailed below.

Dixon scan data were processed to generate quantitative maps of tissue water and fat composition using a MATLAB [24] script written in‐house, taking into account a multi‐spectral model of fat, a single T_2_* component and noise bias correction [25]. TA muscle delineation was performed by manually drawing ROIs on all five slices of the resulting water content images (taking care to exclude intermuscular fat and vascular structures) using ITK‐SNAP (v3.8.0) [26], then applied to calculated fat fraction maps. TA fat fraction was calculated as the average of the TA volume visible in the scan field of view.

### 2.5 Dynamic Spectroscopy Analysis

^1^H PRESS spectroscopy datasets were analysed using the AMARES algorithm of the spectroscopic signal processing package jMRUI (v3.2) [27] to determine water signal amplitude at each TE, from which water ^1^H T_2_ was calculated by fitting a decaying monoexponential function to the dependence of water signal amplitude on echo time. Similarly, post‐exercise T_2_ decline half‐life was measured by fitting a decaying monoexponential function to the change in measured T_2_ across post‐exercise timepoints.

^23^Na‐MR spectroscopy data were exported and analysed in MATLAB. The MRS_lib processing tool [28] was used to read each dataset and determine ^23^Na signal amplitude, all other ^23^Na spectroscopy analysis was performed using MATLAB scripts written in‐house. The half‐life for ^23^Na signal amplitude recovery after exercise (T_1/2_) in the TA was estimated by fitting the recovery time course to a three‐parameter (theoretical post‐exercise ^23^Na signal amplitude at *t* = 0 s, T_1/2_, and baseline ^23^Na signal) exponential decay function to the change in ^23^Na signal with time after cessation of exercise.

^23^Na signal amplitude was measured as peak area in Fourier transformed ^23^Na‐ISIS scan data. ^23^Na T_2_* was measured by fitting a bi‐exponential decay function to each time‐domain ISIS measurement, as shown in Equation (3).

(3) $$\begin{array}{c} M\left(t\right)=M_{\mathrm{fast}}e^{-\frac{t}{T_{2\;\mathrm{fast}}^{*}}}+M_{\mathrm{slow}}e^{-\frac{t}{T_{2\;\mathrm{slow}}^{*}}}+\mathrm{noise} \end{array}$$

where *M(t)* is the magnitude of signal measured at time, *t* (milliseconds), since the centre of the excitation pulse. M_fast_ and M_slow_ are the relative magnitudes of the fast and slow decaying T_2_* components of the ^23^Na signal at *t* = 0. All exponential fitting calculations used a nonlinear least‐squares trust‐region reflective algorithm [29].

### 2.6 Statistical Analysis

Descriptive statistical analysis was applied to demographic and baseline characteristics of both experimental groups. Statistical analysis was performed using IBM SPSS® Statistics software version 21. A two‐tailed Mann–Whitney *U* test was used to test the significance of differences in baseline measures identified in dysferlinopathy patients versus healthy controls in the TA muscle. The Wilcoxon test for repeated measures was used to identify significant changes at baseline and over time after exercise between the groups. Correlation analysis used Spearman correlation coefficient (*r*_*s*_). The results of all statistical studies were considered significant for a *p*‐value lower than 0.05.

## 3 Results

### 3.1 Clinical Characteristics and Baseline Measurements

Dysferlinopathy patients' demographics and clinical characteristics are displayed in Table 2. All patients were ambulant; however, different degrees of muscle weakness were observed in the lower limbs, and upper limb function was within normal limits for all but one patient.

**TABLE 2:** Baseline demographics, clinical characteristics and fat fraction of dysferlinopathy patients.

Pt	Sex	Age at symptom onset	Age at time of study	TA muscle FF (%)	Peak F ankle DF (N)	Pathogenic variant 1	Pathogenic variant 2	CK (U/L)	BMI (kg/m^2^)	NSAD	Ambulation status	10 MWT (s)	100 MWT (min:s)	TUG (s)	PUL	Falls (yearly)
1	Male	36	42	5.9	174	c.4794G > T	c.3277_3279del	2728	29.4	52	Independent	4.6	0m42s	4.6	42	1
2	Female	50	61	21.7	42	c.757C > T	c.855 + 1delG	1235	NA	21	Independent	14.2	2m16s	16.4	31	7
3	Male	13	23	18.7	11	c.4551G > A	c.4551G > A	12 986	21.6	23	Independent	9.8	1m35s	10.6	41	24
4	Female	15	27	32.2	36	c.6056G > A	c.6056G > A	4785	22.7	18	Independent	9.7	1m48s	17.1	42	0
5	Female	28	37	44.4	28	c.2875C > T	c.3149_3150delTC	5568	22.5	26	With aids	9	1m35s	11.1	42	2
6	Female	28	44	26.9	56	c.757C > T	c.757C > T	1825	21.5	11	With aids	13.1	unable	21.4	42	12
7	Female	14	32	15.6^a^	N/A	c.855 + 1delG	c.3031G > C	2694	21.3	38	Independent	6.8	1m10s	6.1	42	0
8	Female	20	44	32.2	14	c.3805dupG	c.5698_5699delAG	2665	24.4	34	With aids	7.8	unable	7.8	42	2
9	Female	24	35	53.7^a^	20	c.525delT	c.5979dupA	4075	25.7	22	Independent	9.6	1m18s	11.9	42	4
10	Female	19	30	5.4^a^	45	c.525delT	c.5979dupA	3778	25.4	32	Independent	6.5	1m1s	8.3	42	18

Abbreviations: BMI: body mass index; CK: creatinine phosphokinase; DF: dorsiflexion; F: force; FF: fat fraction; min: minutes; MWT: metre walk test; N: Newtons; N/A: not available; NSAD: North Star Assessment for Limb Girdle Muscular Dystrophies; PUL: performance upper limb; s: seconds; TUG: time up and go; U/L: units/litre.

^a^ Fat fraction measurements were obtained between 1 and 2 years before the ^23^Na MRI scan. For all other patients, FF measurements were made within 48 h of the ^23^Na scan.

**FIGURE 2:**
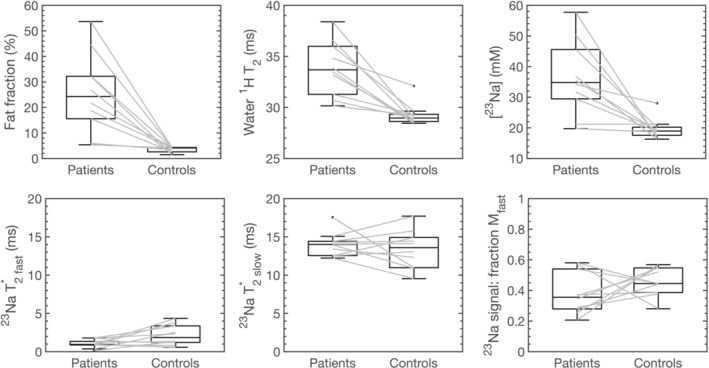
Boxplots depicting baseline (pre‐exercise) quantitative MRI biomarkers in 10 patients with dysferlinopathy and their individually age and sex‐matched healthy controls. ^1^H: hydrogen‐1, ^23^Na: sodium‐23, ISIS: image selected in vivo spectroscopy, M_fast_: fast‐decaying component of the signal magnitude; mM: millimolar, ms: milliseconds, T_2_*: observed transverse relaxation time.

Table 3 shows a summary of multiparametric MR measurements for patients with dysferlinopathy and for healthy controls at baseline and post‐exercise. Figure 2 plots the differences in baseline multiparametric MR measurements between patients and their age‐ and sex‐matched healthy controls. The patient group exhibited a significantly higher TA fat fraction and a lower peak force during ankle dorsiflexion. Baseline MR measurements showed statistically significant differences between healthy volunteers and dysferlinopathy patients; TA sodium concentration was, on average, 80% higher in patients with dysferlinopathy than in healthy volunteers, and ^1^H water T_2_ was 15% higher in patients compared to healthy volunteers. The inter‐scan repeatability of the ^23^Na‐ISIS acquisition was tested over three repeated acquisitions in a subset of participants, where baseline ^23^Na‐ISIS signal intensity was found to have an inter‐scan standard deviation of 3.1%.

**TABLE 3:** Summary of pre‐ and post‐exercise MR measurements in dysferlinopathy patients and their matched healthy controls.

	Dysferlinopathy patients (*n* = 10)	Healthy controls (*n* = 10)	*p* (two‐tailed)
Participant characteristics			
Sex			
Female/male	8/2	8/2	Matched
Age (years)			
Mean	38.0	38.9	0.94
SD	10.8	11.5	
TA muscle FF (%)			
Mean	25.7	3.4	< 0.001
SD	15.6	1.2	
Peak force ankle dorsiflexion (*N*)			
Mean	47.3	104.2	0.005
SD	49.7	25.2	
Pre‐exercise MRI			
^1^H water T_2_ (ms)			
Mean	33.8	29.3	< 0.001
SD	2.7	1.1	
^23^Na‐ISIS signal			
Mean	1.29 × 10^−4^	8.7 × 10^−5^	0.023
SD	4.8 × 10^−5^	3.3 × 10^−5^	
^23^Na‐radial SNR			
Mean	76	44	0.019
SD	32	22	
^23^Na‐cartesian SNR			
Mean	27	11	0.001
SD	10	4	
TA sodium concentrations (mM)			
Mean	36.2	19.6	< 0.001
SD	11.4	3.1	
Post‐exercise MRI			
Peak ^1^H water T_2_ (ms)			
Mean	36.2	36.4	0.94
SD	3.2	3.4	
Peak ^23^Na‐ISIS signal			
Mean	1.4 × 10^−4^	1.1 × 10^−4^	
SD	4.3 × 10^−5^	3.0 × 10^−5^	0.08

Abbreviations: ^1^H: hydrogen; FF: fat fraction; ISIS: image selected in vivo spectroscopy; ms: milliseconds; N: Newtons; SD: standard deviation; SNR: signal to noise ratio; TA: tibialis anterior.

### 3.2 ^23^Na‐MR Imaging and Spectroscopy

**FIGURE 3:**
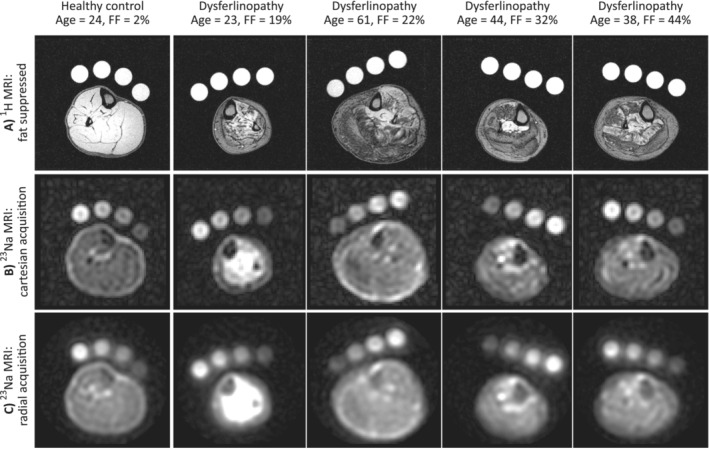
(A) ^1^H‐MR images, (B) ^23^Na‐MR images taken using a cartesian acquisition sequence and (C) ^23^Na‐MR images taken using a radial stack of stars imaging sequence, depicting equivalent image slice positions. An image set from a representative healthy volunteer is presented beside four dysferlinopathy patient image sets, where varying degrees of fat infiltration in the tibialis anterior muscle were measured. ^1^H: hydrogen‐1, ^23^Na: sodium‐23, FF: fat fraction, MRI: magnetic resonance imaging.

Figure 3 shows ^23^Na‐MRI and fat‐supressed ^1^H‐MRI data from a healthy volunteer and from four of the patients with dysferlinopathy. ^1^H‐MRI scans show the variation in muscle replacement by fat across the cohort, where brighter muscle tissue is replaced by darker lipid in some muscle groups of some patients. ^23^Na‐MR images were acquired with both cartesian (TE = 1.89 ms) and radial (TE = 0.28 ms) acquisitions and showed a variable extent of elevation in sodium content across the patient cohort compared to healthy controls, with no overt dependence on the degree of muscle replacement by fat.

### 3.3 Post‐Exercise MRI

**FIGURE 4:**
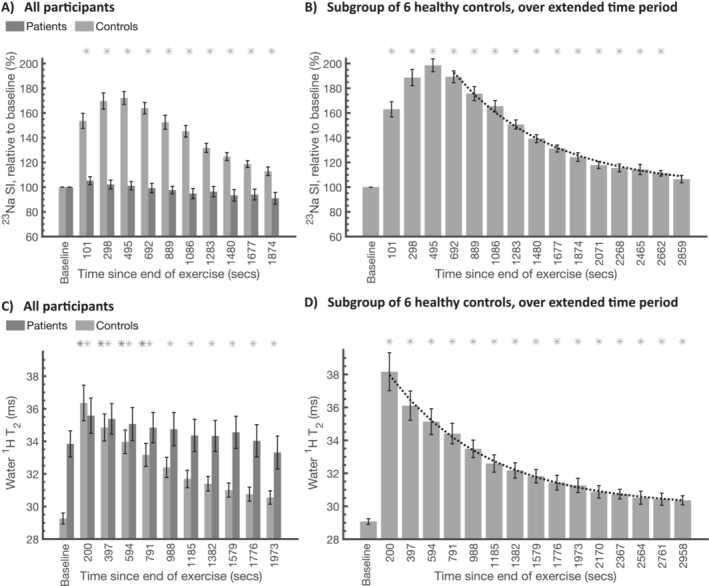
Dynamics of ^23^Na signal amplitude (A and B) and water ^1^H T_2_ (C and D) in patients and healthy volunteers at baseline, and after exercise. Asterisk (*) denotes a significant deviation from baseline (two‐tailed, *p* < 0.05) for healthy controls (light grey asterisk) or patients (dark grey asterisk). Error bars denote the standard error on the mean. Subplots A and C present data collected over a ~35‐min time period post‐exercise in all participants. Subplots B and D display equivalent data collected over a longer post‐exercise period from a subgroup of six healthy controls, presented here for completeness. ^1^H: hydrogen‐1, ^23^Na: sodium‐23, ms: milliseconds; secs: seconds, SI: signal intensity, T_2_: transverse relaxation time.

Figure 4 shows the post‐exercise dynamics of muscle water ^1^H T_2_ and muscle ^23^Na signal amplitude. In healthy volunteers the sodium signal amplitude rose during the initial ~8 min post‐exercise and returned to baseline over 30 min post‐exercise. In the subset of 6 healthy controls where data was collected for a longer duration post‐exercise the recovery to baseline had a half‐life of 636 s (95% CI = 49 s) (Figure 4B). Dynamic changes in ^23^Na‐ISIS signal amplitude in the patient group post‐exercise were highly variable between participants. On average there was a post‐exercise increase in ^23^Na signal amplitude, but this increase compared to baseline was not statistically significant and remained relatively stable over the post‐exercise observation period time with a small downward trend (Figure 4A). Muscle water ^1^H T_2_ values were substantially elevated from the first post‐exercise timepoint in both groups (Figure 4C) and did not show the progressive rise in post‐exercise ^23^Na signal amplitude observed over the first 5–10 min, prior to return toward baseline. Post‐exercise elevation in water ^1^H T_2_ in healthy controls showed a recovery to baseline values (Figure 4C,D) with a half‐life of 620 s (95% CI = 90 s). The post‐exercise dynamics of water ^1^H T_2_ in patients was again heterogenous and minimal compared to healthy volunteers (Figure 4C).

### 3.4 ^23^Na T_2_*

**FIGURE 5:**
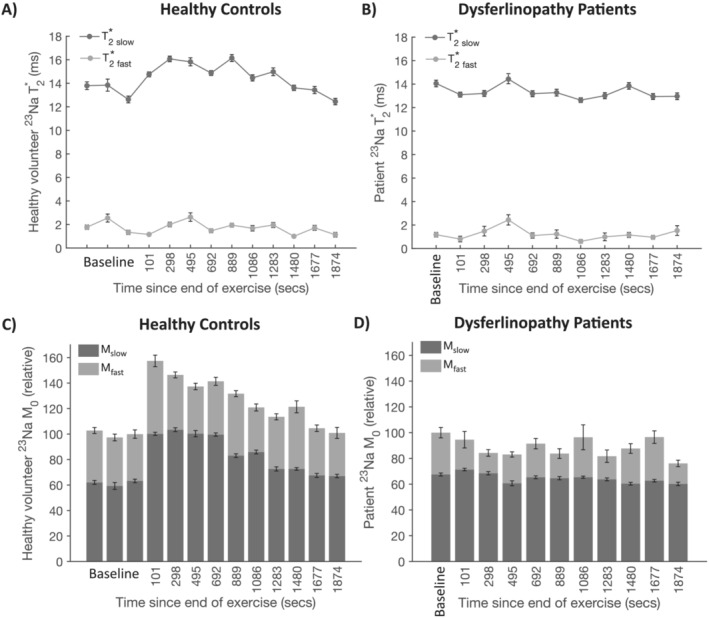
Dynamic ^23^Na fast‐ and slow‐decaying components of the biexponential T_2_* values at baseline and after exercise in (A) healthy controls and (B) patients with dysferlinopathy. The corresponding relative amplitudes, M_fast_ and M_slow_, in (C) healthy controls and (D) patients with dysferlinopathy. ^23^Na: sodium‐23, M_fast_: signal magnitude of the fast‐decaying component of the T_2_* at time = 0, M_slow_: signal magnitude of the slow‐decaying component of the T_2_* at time = 0, ms: milliseconds; secs: seconds; T_2_*_fast_: fast‐decaying component of the T_2_*, T_2_*_slow_: slow‐decaying component of the T_2_*.

^23^Na T_2_* relaxation rate was assessed by quantitation of fast‐ and slow‐relaxing components, pre‐ and dynamically post‐exercise (Figure 5). At baseline, the T_2_*_slow_ was 13.4 ms (SD: 2.3 ms), and T_2_*_fast_ was 2.2 ms (SD: 1.3 ms) in healthy controls (Figure 4A), whereas in patients the T_2_*_slow_ was 14.0 (1.5) ms and T_2_*_fast_ was 1.0 (0.5) ms (Figure 4B). At baseline, the fraction of ^23^Na signal with fast‐decaying T_2_ was 46% (SD: 9%) in healthy controls and 38% (SD: 13%) in patients (Figure 4C,D). The magnitude of the slow‐relaxing component, M_slow_, showed a transient and statistically significant increase from baseline levels after exercise in the healthy controls' cohort, as did T_2_*_slow_. Figure 5A,C shows that the post‐exercise evolution of ^23^Na signal intensity is dominated by change in M_slow_ and T_2_*_slow_, which both show a progressive post‐exercise increase. In contrast, the average total ^23^Na signal amplitude began recovery immediately post‐exercise, analogous to the time course of change in post‐exercise water ^1^H water T_2_*. No statistically significant change in M_fast_ and T_2_*_fast_ was observed in healthy volunteers (Figure 5A,C), and no statistically significant changes in ^23^Na T_2_* component magnitudes or relaxation rates were observed in patients with dysferlinopathy (Figure 5B,D).

### 3.5 Correlation Analysis

No correlations were found between age, fat fraction in the TA muscle and peak force achieved when dorsiflexing the ankle and MR parameters in either cohort.

## 4 Discussion

We have applied dynamic volume‐localised ^23^Na spectroscopy to attain high temporal resolution measurements of muscle sodium dynamics in patients with dysferlinopathy and in matched healthy controls. We have characterised muscle sodium content at rest and dynamically post‐exercise. Dysferlinopathy patients showed significantly higher baseline water ^1^H T_2_ and higher baseline sodium concentrations than controls. Moreover, we observed that muscle water ^1^H T_2_ and sodium content increased significantly after an isometric exercise in healthy controls, with a progressive return to baseline levels. These changes were not observed in patients with dysferlinopathy.

Skeletal muscle is primarily composed of muscle fibres (over 90% of the tissue mass) and stroma (comprising connective tissue and blood vessels). In dysferlinopathy, there is a progressive loss of muscle fibres and an increase in stromal content due to an expansion of fibrotic and fat tissue, alongside an increase in inflammatory cells. It is well established that dysferlin deficiency leads to a defect in the fibres' ability to seal the muscle membrane after damage, permitting unregulated entry of diverse molecules, including calcium and sodium ions, into the intracellular space [30]. These physiopathological changes may contribute to increased baseline sodium concentrations observed in patients with dysferlinopathy, as well as elevation in water ^1^H T_2_, which is associated with active muscle fibre damage such as inflammation or necrosis [31, 32]. Our baseline findings also align with ^23^Na‐MRI results reported in studies of patients with Duchenne muscular dystrophy and facioscapulohumeral dystrophy, where the muscle fibre membrane is fragile and prone to damage after muscle contraction [14, 15].

Repeated interleaved ^23^Na and ^1^H spectroscopic scans permitted synchronous characterisation of post‐exercise sodium and ^1^H water T_2_ dynamics. A statistically significant increase in ^23^Na signal intensity and in ^1^H water T_2_ was observed in healthy volunteers immediately following exercise. These results align with findings reported previously in the literature [33, 34]. However, while ^1^H water T_2_ values peaked at the first post‐exercise timepoint, the ^23^Na signal intensity was maximal at approximately 8 min after completion of exercise. To our knowledge, this difference has not previously been reported. The current literature of muscle dynamic ^23^Na‐MRI describes delays between cessation of exercise and commencement of ^23^Na‐MR acquisitions (approx. 7 and 15 min in references [10, 13], respectively) and a substantially lower temporal resolution of the image‐based dynamic ^23^Na‐MRI scans that may have rendered studies insensitive to the initial rise and plateau of sodium signal amplitude prior to the subsequent recovery towards baseline values. The higher temporal resolution measurements used in this study, afforded by a spectroscopic rather than imaging‐based approach, permitted a more detailed visualisation of the dynamic ^23^Na signal time course.

Post‐exercise elevation of water ^1^H T_2_ has previously been attributed to an increase in intracellular water content [35] and intramuscular metabolite accumulation [36] during exercise, which progressively return to baseline values. The temporal discrepancy between maximal ^1^H T_2_ and maximal ^23^Na signal intensity suggests that the ^23^Na signal is governed by different biophysical mechanisms after the culmination of exercise which do not impact water T_2_. Patients with dysferlinopathy did not exhibit a significant increase in either water ^1^H T_2_ or ^23^Na signal intensity following exercise. This may reflect the substantial elevation of both water ^1^H T_2_ and ^23^Na content in patients at baseline compared to controls. Alternatively, patients may not have adequately exercised the TA muscle due to weaker dorsiflexors, resulting in the two experimental groups necessarily exhibit substantial differences in maximum achievable force. We attempted to mitigate this by having an expert physiotherapist [M.R. or D.M.] present at all study sessions to ensure close adherence to the exercise protocol.

One of the 10 patients had a TA FF% close to the values measured in the healthy controls at the time of the ^23^Na scan. This patient exhibited baseline ^23^Na content and water ^1^H T_2_ values that matched the control group, and the dynamic behaviour of muscle ^23^Na content and water ^1^H T_2_ after exercise showed a pattern similar to that of healthy volunteers. Similar findings have been described for patients with facioscapulohumeral muscular dystrophy [15], whereas a study of patients with Duchenne muscular dystrophy reported elevated sodium content even in muscles without substantial elevation of fat content [14]. The study of muscle sodium content and exercise‐induced sodium dynamics in pre‐symptomatic patients has potential to determine whether ^23^Na‐MRI can detect and report on pathophysiological changes upstream of gross muscle structural change (i.e., replacement of muscle fibres by fat).

The multicomponent nature of ^23^Na T_2_* relaxation arises from transient interactions between tissue molecular electric field gradients and the electric quadrupolar moment of ^23^Na nuclei [17]. In homogenous environments where ^23^Na nuclei are in a motion‐restricted regime, these interactions result in T_2_* relaxation with fast‐ and slow‐relaxing components, whereas ^23^Na nuclei in an unrestricted aqueous environment exhibit slow T_2_* relaxation. The post‐exercise increase in ^23^Na T_2_* observed in our study was principally due to an increase in the volume of slow‐decaying T_2_*, M_slow_ (Figure 5), suggesting a greater proportion of sodium in a dilute environment. Such a change is consistent with transient oedema. Fluctuations in M_fast_ following exercise were also present, and appeared to follow a different post‐exercise recovery timecourse to M_slow_, suggesting sensitivity to changing content of sodium ions in non‐dilute compartments via a different mechanism.

Volume localisation by the ISIS method is well‐suited to ^23^Na‐MR applications. Sodium nuclei have an in vivo T_1_ relaxation rate (15–55 ms at 3T [16]) that is sufficiently long to retain magnetization within the timescale of the three inversion pulses required for volume localisation, yet it is adequately short to permit near‐complete relaxation within a short repetition time (162 ms in our studies) that permits a high degree of signal averaging (and consequent SNR improvements) in short duration scans. SNR is also influenced by voxel dimensions: The in‐plane dimensions of the ^23^Na‐ISIS voxel size was defined on a per‐participant basis as it was limited by muscle dimensions, most strikingly in the patient cohort. Studies in larger proximal limb muscle groups offer the opportunity to acquire ^23^Na‐MR data at a substantially higher temporal resolution, though they may be more challenging to fatigue in situ. Nonetheless, ISIS volume‐localised ^23^Na‐MR methods may serve as a valuable tool in studies of muscular dystrophy as well in other organs (such as neuroimaging) where there is ongoing interest in probing the multiexponential nature of ^23^Na‐T_2_* relaxation in health [37, 38] and in disease states [39, 40].

The main limitations of this study were the small number of participants (although this is a relatively rare disease) and the heterogeneity in the amount of fat within the TA muscle, despite all patients being ambulant and capable of achieving dorsiflexion against gravity. A larger and more phenotypically diverse cohort, including more asymptomatic and mildly symptomatic patients, would improve our understanding of the distribution of ^23^Na signal intensity and T_2_* properties across the disease spectrum.

In conclusion, we present a new approach to study sodium muscle concentration and its changes after exercise by using a widely available MRS technique. Our findings demonstrate that ^23^Na‐ISIS MR presents a valuable and time‐efficient methodology for dynamic spatially localised measurement of tissue sodium content and T_2_* relaxation properties. To our knowledge, our data represents the first description of increased muscle sodium content in a cohort of patients with dysferlinopathy. Our studies demonstrated higher sodium content in muscles of dysferlin‐deficient patients compared to controls, suggesting that dystrophic changes in skeletal muscle tissue lead to an increase in sodium, independent of the degree of fat replacement. The dynamics of muscle sodium content after exercise showed an increase in healthy controls that was not present in dysferlin‐deficient patients. Our results support the use of ^23^Na‐MRI in the study of healthy muscle response to exercise, which could guide the design and use of exercise programs in healthy individuals or aged patients with sarcopenia. ^23^Na‐MR spectroscopy and imaging have potential for use as an early marker of muscle damage in muscular dystrophies when pathophysiological change results in increased muscle sodium content and/or an abnormal response to exercise.

## Ethics Statement

The authors certify that they have complied with the ethical guidelines for authorship and publishing in the *Journal of Cachexia, Sarcopenia and Muscle*.

## Conflicts of Interest

Kieren G. Hollingsworth undertakes consultancy and contract research with Astellas Gene Therapies on neuromuscular diseases not related to the present manuscript. Volker Straub has served on advisory boards for Astellas Gene Therapies, Biogen, Edgewise Therapeutics, Ipsen, Kate Therapeutics, ML Bio Solutions, Novartis Gene Therapies, PepGen, Roche, Sanofi, Sarepta Therapeutics, Vertex Pharmaceuticals and Wave Therapeutics. He has received speaking fees/honoraria from Novartis Gene Therapies, Pfizer, Roche, Sanofi and Sarepta Therapeutics and has received grants for clinical research from Sarepta Therapeutics and Sanofi. Mary A. Neal, Carla F. Bolano‐Diaz, Mark Richardson, Jassi Michell‐Sodhi, Robert Muni‐Lofra, Meredith K. James, Heather Hilsden, Ian Wilson, Andrew M. Blamire, Peter E. Thelwall and Jordi Diaz‐Manera have no conflicts of interest to declare.
